# Unfolding COVID-19 vaccine communication campaigns in China’s neighborhoods: a qualitative study of stakeholders’ narratives

**DOI:** 10.3389/fpubh.2023.1253844

**Published:** 2023-11-30

**Authors:** Ronghui Yang, Yanchao Han

**Affiliations:** School of Humanities, Donghua University, Shanghai, China

**Keywords:** COVID-19 vaccine, communication campaign, neighborhood, political pressure, continuity

## Abstract

**Introduction:**

The Chinese state has recently implemented the COVID-19 Vaccine Communication Campaign (CVCC) to counter vaccine hesitancy. Nonetheless, the extant literature that examines COVID-19 vaccine acceptance has less represented COVID-19 vaccine communication efforts.

**Methods:**

To address this lacuna, we qualitatively explored how CVCCs were organized in Chinese communities by investigating 54 Chinese stakeholders.

**Results:**

This study indicates that the CVCC was sustained by top-down political pressure. CVCCs’ components involve ideological education among politically affiliated health workers, expanding health worker networks, training health workers, implementing media promotion, communicating with residents using persuasive and explanatory techniques, encouraging multistakeholder partnerships, and using public opinion-steered and coercive approaches. While CVCCs significantly enhanced COVID-19 vaccine acceptance, lacking open communication, stigmatizing vaccine refusers, insufficient stakeholder collaboration, and low trust in the COVID-19 vaccination program (CVP) eroded the validity of CVCCs.

**Discussion:**

To promote the continuity of CVCCs in China, CVCC performers are expected to conduct open and inclusive communication with residents. Furthermore, CVP planers should create robust partnerships among health workers by ensuring their agreements on strategies for implementing CVCCs and optimize COVID-19 immunization service provision to depoliticize CVPs. Our study will not only deepen global audiences’ understanding of CVCCs in authoritarian China but also offer potential neighborhood-level solutions for implementing local and global public health communication efforts.

## 1 Introduction

The COVID-19 vaccination plays a vital role in containing virus spread, protecting personal health, and preventing the collapse of healthcare systems and the economy from shutting down to contain the pandemic outbreak (1). To roll out the COVID-19 vaccination program (CVP) across the country, the Chinese state created a series of policies, such as “Technical Guidelines for COVID-19 Vaccination” issued in March 2021, “Notice on Further Optimizing the Implementing of COVID-19 Prevention Measures” and “Implementation Plan for Second Dose of COVID-19 Vaccine” issued in December 2022, and “Vaccination Work Plan for Response to Recent COVID-19 Infection” issued in April 2023 (2), to provide policy support for COVID-19 vaccinations.

In academia, mainstream scholarship related to COVID-19 vaccination centers on the willingness, attitudes, confidence, and acceptance of the COVID-19 vaccine among different groups, such as healthcare workers, patients living with chronic diseases, college students, older adults, and vaccine hesitators, and their determinants (3, 4), and strategies for implementing CVPs, such as social campaigns, incentives, and science popularization (5, 6). However, COVID-19 vaccine communication has been less explored in China. In reality, influenced by the principle of “informed, consented and voluntary” created by the central state in 2022, many citizens chose not to be vaccinated or not to be fully vaccinated. To swiftly achieve a high vaccination rate and reach herd immunity as quickly as possible to contain virus spread, local states implemented COVID-19 vaccine communication efforts to deter vaccine misinformation, eliminate citizens’ negative attitudes toward COVID-19 vaccination, and address vaccine hesitancy (7, 8). Informed by these analyzes above, we explore how COVID-19 vaccine communication programs are organized in China.

To further confirm the research lacuna, we traced academic debates on vaccine communication activities in China. Extant research has primarily explored the routine science-based popularization of vaccines, doctor–patient communication on vaccines and patients, and risk communication during vaccine incidents. For instance, Yang et al. (9) indicated that many Chinese citizens with insufficient scientific literacy are easily misled by vaccine disinformation, necessitating vaccination science popularization among citizens. Therefore, Ren and Zhai (10) and Li et al. (11) explored diverse media tools of science popularization, such as speeches, broadcasts, exhibitions and periodicals, magazines, mass media, and media convergence. Hou et al. (12) and Hu et al. (13) demonstrated that doctor–patient communication on vaccines helps to significantly increase vaccine acceptance since physicians possess professional knowledge and their recommendations are considered by the public to be reliable. Following a vaccine crisis, governments should carry out risk communication with citizens swiftly and maintain transparency to reduce the negative consequences caused by this crisis and regain public confidence in vaccination (1, 14). Therefore, Ma et al. (15) argued that governments must accurately identify and respond to public demands and sentiments to dispel fear and anxiety in vaccine crises. To ensure the reliability of information sources, authoritative professionals should steer risk communication following vaccine crises (16). In addition, given that a single stakeholder is generally unable to effectively address fragmented public needs, creating a multi-stakeholder partnership in risk communication during vaccine incidents is also necessary (17).

In summary, previous studies have explored strategies for implementing immunization promotion efforts in routine times, risk communication during vaccine incidents, and doctor–patient interactions in informing vaccination hesitancy. However, these studies have not focused on campaign strategies for implementing vaccine communication programs in emergencies. The mobilization campaign, which originated in military affairs, refers to the launch of a series of actions or events launched to gain public support and achieve a particular goal (18). The elements of a campaign involve creating campaign goals, defining and engaging with target audiences, offering key information that induces changes, and distributing campaigns via multiple media (19). In the context of public health emergencies, such as epidemics and widespread vaccine hesitancy, conventional means fail to effectively promote public acceptance of vaccines. In this case, deploying an immunization campaign helps create a favorable information environment, reach a wide audience, boost vaccine acceptance, and swiftly achieve vaccination goals. As such, the Chinese state launched the COVID-19 Vaccine Communication Campaign (CVCC) in neighborhoods to convince citizens to accept COVID-19 vaccines.

Therefore, we explored how CVCCs were organized in communities. Specifically, this study interrogated the drivers, strategies, and vulnerabilities of CVCCs in Chinese communities. Investigating CVCCs in China helps identify the specific risks associated with CVCCs and offers countermeasures to increase the resilience of CVCCs in China. These findings could also enrich international debates about COVID-19 vaccine communication, deepen global audiences’ understanding of COVID-19 vaccine communication in an authoritarian regime, and offer potential neighborhood-level solutions for implementing local and global public health communication efforts. In the following sections, we present the methods through which we achieved this and their results, discuss the findings, and offer contributions that can inform policy agenda setting in China and international debates.

## 2 Methodology

### 2.1 Research design

In this study, we adopted qualitative methods to gain insight into the scenarios, elements, and risks of CVCCs in Chinese communities. We conducted semi-structured interviews with Chinese stakeholders to gather data in an exploratory way. We interviewed respondents via face-to-face interaction, telephone, and video calls on WeChat (akin to WhatsApp) between July 2022 and December 2022. To incentivize participation, we offered gifts to respondents interviewed offline and 50–150 RMB (6.56–19.67 €) to those interviewed remotely. Participant recruitment procedures were aligned to the specific Chinese context: selection started through informal, personal networks, and continued through snowballing to include participants’ colleagues. For instance, we first interviewed staff of the resident’s committee we were familiar with and recruited more participants via referrals from acquaintances. Subsequently, we identified other potential subjects via these enrolled participants. These sampling methods generated enough data for analysis. Prior to the interviews, we obtained oral informed consent from participants after sharing with them the research goals, methods, expected outcomes, anticipated impacts, and rights and responsibilities of participants and after ensuring their anonymity.

### 2.2 Data collection and analysis

We interviewed 54 respondents in Beijing, Guangzhou, Shanghai, Wuhan, and Changshang to reach data saturation (20). The research population included 5 directors, 5 secretaries, 10 staff of the neighborhood committee, 6 doctors in the community public healthcare center, 17 community residents, and 6 members of the homeowner association. These participants were approached because of their knowledge of and experiences with CVCCs (Table 1).

**Table 1:** Participants’ demographic characteristics.

Participant ID	Description of function	Gender	Location
ID1	Director of Yanxi resident’s committee	Female	Shanghai
ID2	Director of Baohuiyuan resident’s committee	Male	Beijing
ID3	Director of Baiyue resident’s committee	Male	Guangzhou
ID4	Director of Huiyuetiandi resident’s committee	Female	Wuhan
ID5	Director of Runzeyuan resident’s committee	Male	Changsha
ID6	Secretary of Yanxi resident’s committee	Female	Shanghai
ID7	Secretary of Baohuiyuan resident’s committee	Male	Beijing
ID8	Secretary of Beiyue resident’s committee	Male	Guangzhou
ID9	Secretary of Huiyuetiandi resident’s committee	Female	Wuhan
ID10	Secretary of Runzeyuan resident’s committee	Male	Changsha
ID11	Staff in Yanxi resident’s committee	Female	Shanghai
ID12	Staff in Yanxi resident’s committee	Female	Shanghai
ID13	Staff in Baohuiyuan resident’s committee	Male	Beijing
ID14	Staff in Baohuiyuan resident’s committee	Female	Beijing
ID15	Staff in Beiyue resident’s committee	Male	Guangzhou
ID16	Staff in Baiyue resident’s committee	Female	Guangzhou
ID17	Staff in Huiyuetiandi resident’s committee	Female	Wuhan
ID18	Staff in Huiyuetiandi resident’s committee	Male	Wuhan
ID19	Staff in Runzeyuan resident’s committee	Male	Changsha
ID20	Staff in Runzeyuan resident’s committee	Male	Changsha
ID21	Resident in Yanxi community	Female	Shanghai
ID22	Resident in Yanxi community	Male	Shanghai
ID23	Resident in Yanxi community	Female	Shanghai
ID24	Resident in Meishumingjia community	Female	Shanghai
ID25	Resident in Meishumingjia community	Male	Shanghai
ID26	Resident in Baohuiyuan community	Male	Beijing
ID27	Resident in Baohuiyuan community	Male	Beijing
ID28	Resident in Baohuiyuan community	Male	Beijing
ID29	Resident in Baiyue community	Male	Guangzhou
ID30	Resident in Baiyue community	Female	Guangzhou
ID31	Resident in Baiyue community	Female	Guangzhou
ID32	Resident in Huiyuetiandi community	Female	Wuhan
ID33	Resident in Huiyuetiandi community	Male	Wuhan
ID34	Resident in Huiyuetiandi community	Female	Wuhan
ID35	Resident in Runzeyuan community	Male	Changsha
ID36	Resident in Runzeyuan community	Male	Changsha
ID37	Resident in Runzeyuan community	Male	Changsha
ID38	Doctor in Changning district public healthcare center	Female	Shanghai
ID39	Doctor in Xinjinzhen community public healthcare center	Male	Shanghai
ID40	Doctor in Baohuiyuan community healthcare center	Male	Beijing
ID41	Doctor in Baiyue Community Healthcare center	Male	Guangzhou
ID42	Doctor in Manchun street Healthcare center	Female	Wuhan
ID43	Doctor in Yuelu district public healthcare center	Male	Changsha
ID44	Property manager in Yanxi community	Female	Shanghai
ID45	Property manager in Baohuiyuan community	Male	Beijing
ID46	Property manager in Baiyue community	Male	Guangzhou
ID47	Property manager in Huiyuetiandi community	Female	Wuhan
ID48	Property manager in Runzeyuan community	Male	Changsha
ID49	Staff of homeowner association in Yanxi community	Female	Shanghai
D50	Staff of homeowner association in Meishumingjia community	Female	Shanghai
ID51	Staff of homeowner association in Baohuiyuan community	Male	Beijing
ID52	Staff of homeowner association in Baiyue community	Male	Guangzhou
ID53	Staff of homeowner association in Huiyuetiandi community	Female	Wuhan
ID54	Staff of homeowner association in Runzeyuan community	Male	Changsha

We did not record the interviews at the respondents’ requests and avoided taking notes in front of participants to mitigate their guardedness and encourage them to express their opinions freely. Gathering data without records are considered unconventional, but we created detailed transcriptions from memory immediately afterwards. Meanwhile, to ensure that we would do justice to original intentions and connotations and the correctness of quotes, in some cases we verified these via WeChat (9). Subsequent thematic analysis was conducted to inductively analyze the transcripts of the interview. Specifically, we first coded the data relevant to the research questions using a semantic approach to gain a condensed overview of the main points that recur throughout the data. Next, we identified sub-themes among these codes and reviewed these sub-themes to ensure their accurate representations of the data. Subsequently, we conceptualized themes among these sub-themes. Finally, to conduct a credible qualitative analysis, two professionals engaged in reviewing each phase of thematic analysis. In that way, sub-themes we identified in this study include the dynamics of CVCCs’ emergence, the driving forces of CVCCs’ continuity, ideological education among politically affiliated health workers, expanding health worker networks, training health workers, implementing media promotion, confirming communication tactics between community health workers and residents, and identifying risks in CVCCs. Important themes identified were the dynamics of CVCCs’ development, organizations, and the risks of CVCCs. Finally, we selected exemplary data extracts from the key themes for inclusion as quotes (21). This qualitative study has followed standards for reporting qualitative research.

## 3 Results

### 3.1 CVCCs in the neighborhood

In this section, we presented the scenario of CVCCs, the dynamics of CVCCs’ emergence, and the continuity and risks in CVCCs.

#### 3.1.1 Scenario of community CVCCs

In November 2022, in a community in Shanghai, we observed that a group of community health workers, wearing uniform red jackets and holding COVID-19 vaccination brochures, were actively communicating with residents to promote the benefits of vaccination. They informed residents of the procedures, sites, and times of COVID-19 vaccination, promoted the benefits of vaccination, discussed vaccination precautions, and taught residents how to make an appointment for COVID-19 vaccination via the workers’ vaccination service application on WeChat. Several community health workers were also posting on community bulletin boards and hanging up banners at the entrance to the community to advertise the importance and urgency of the COVID-19 vaccination. A cluster of health workers, led by the secretary of the neighborhood committee, visited residents’ homes to investigate their attitudes toward vaccination, address their vaccine concerns, and encourage residents eligible for vaccination to get the COVID-19 vaccine. Furthermore, some community health workers communicated with residents who were waiting in line for COVID-19 vaccination at the community site about precautions after vaccination. COVID-19 vaccination became a main theme of Chinese neighborhood governance in 2022, and sparked massive media coverage.

The foregoing scenario reflects a typical Chinese COVID-19 vaccination campaign in a neighborhood. These campaigns were not exclusive to certain communities or neighborhoods; instead, they occurred in most urban communities in China (ID1-4). The CVCC, a key component of the COVID-19 vaccine campaign involving top-level policy designs and grassroot-level policy implementation, refers to various activities health workers carry out with target audiences. Health workers addressed vaccine-related concerns to change citizens’ knowledge, beliefs, opinions, and behavior regarding vaccination in the direction desired by these health workers.

#### 3.1.2 Dynamics of community CVCCs’ development

In this section, we explore the dynamics of CVCC’s emergence and continuity. The emergence of CVCCs is attributed to the advantage of social campaigns in reaching vaccination goals, to the voluntary vaccination policy, and to state reliance on the campaign paradigm in authoritarian China. Compared to routine strategies, mobilization campaigns help prioritize CVPs, tweak the bureaucratic system, and accumulate resources underpinned by the principle of handling special matters with special arrangements to achieve political goals effectively (ID 5–9). Under the principle of voluntary vaccination, mandatory measures are more likely to induce media exposure and public criticism, damage the government’s reputation, and trigger public accountability (ID 10–11). The social mobilization approach encourages voluntary vaccination via tactical communications with citizens, aligning with the voluntary immunization policy. Additionally, given the successful experience of social mobilization in the past and the current political structure, the Chinese government would conventionally deploy mobilization campaigns to address complex governance problems after routine means fail (22).

The continuity of CVCCs is attributed to top-down political pressure. CVCCs that emphasize extensive tweaks to the bureaucratic system cannot be sustained in the long run without external forces (ID 17–20). To ensure the continuity of CVCCs, the top-level government constantly exerts pressure on lower-level states via a series of measures such as accountability, incentives, and political mobilization to promote states’ active performance of duties and efficiently achieve political goals. Political pressures in relation to the COVID-19 vaccine campaign were exerted with the aim of achieving a high vaccination rate. Therefore, the central state conceptualized COVID-19 vaccination as a “major political task” in April 2022 and adopted a series of measures, such as setting specific goals, issuing various policies, and intensifying the supervision and accountability of local officials, to stimulate the implementation of CVPs at the local level (ID 1–3). One respondent we interviewed argued that,

In 2022, the central government has promulgated a total of 18 vaccination-related policies, and inspection teams delegated by the central state conducted more than 100 inspections of vaccination work in local areas (ID 1).

Correspondingly, local governments outlined the responsibilities of various grassroot-level government agencies, conducted political mobilization among local officials, and established accountability and incentive mechanisms to stimulate healthcare workers to actively perform their duties (ID 8). Under top-down pressure, community healthcare workers initiated CVCCs to increase residents’ acceptance of vaccination (ID 24).

### 3.2 Organization of community CVCCs

The elements of CVCCs in the community involve conducting ideological education among politically affiliated health workers, expanding the network of health workers, training health workers, and communication between health workers and residents.

#### 3.2.1 Ideological education among politically affiliated health workers

In an authoritarian regime, ideological education refers to a social practice in which states or social groups indoctrinate political ideas, beliefs, and moral norms to improve their political identity and compliance, enhance social solidarity, and enable the bureaucratic system to manage threats (23). During the CVCCs, the party-state carried out intensive ideological education among politically affiliated health workers composed of members of the neighborhood committee and of the Chinese Communist Party (CCP) branch in the community to improve their political identity with and loyalty to the CCP. These educational campaigns also enhanced health workers’ sense of serving the people and ensured the pragmatic implementation of COVID-19 vaccination policies. Meanwhile, ideological education also helped eliminate disagreements and conflicts among politically affiliated health workers, create a consensus on strategies for implementing CVPs among them, and increased their sense of solidarity and collaboration (ID 9, 21).

In practice, community managers performed ideological education activities by holding mobilization meetings and CCP meetings, inviting senior officials to engage in community-based activities, and conducting COVID-19 vaccination online education programs. Specifically, neighborhood committee leaders frequently organized vaccination mobilization meetings to convey the spirit and intent of the central leadership’s important speeches on COVID-19 vaccination and to organize politically affiliated health workers to study COVID-19 vaccine-related policy documents, aiming to deepen their understanding of agreement with these vaccination policies (ID 16–19). One of the our respondents indicated that,

The secretary of the neighborhood committee announced at the community mobilization meeting: We must thoroughly perform COVID-19 vaccination tasks assigned by the higher-level government, enhance our sense of political responsibility, adhere to the strategy of “people first, life first” and the principle of “ensuring all people eligible for vaccination have access to it” created by the central state, and do our damnedest to implement vaccine communication activities (ID 18).

Senior officials, such as leaders of the municipal government and the sub-district office, were also invited to participate in the community mobilization meetings to provide institutional support for CVPs, signaling that senior governments attached great importance to CVPs. These who perfunctorily implemented CVPs were considered disloyal and disobeying a superior’s orders, and they will be accountable (ID 4, 5). Community CCP branches regularly held meetings as well, encouraging their members to practice self-reflection on their previous immunization work, and learn the CCP’s principles and regulations and COVID-19 immunization policies, reinforcing CCP members’ political obedience and awareness of serving the people, and ensure standardized implementation of CVPs. These efforts shaped CCP members’ initiatives in implementing community CVCCs (ID 9–11).

#### 3.2.2 Expanding health worker networks

Merely relying on state forces cannot lead to successfully implementing CVCCs, and partnering with multiple stakeholders is therefore necessary. In doing so, neighborhood committees expanded health worker networks by absorbing members of the grid-based governance system, including property managers, members of homeowner associations and healthcare practitioners of the community public health service center. They also recruited volunteers (ID1-3, 11–14). A director of the neighborhood committee argued that,

The grid management model has been widely deployed by governments to optimize public service provision in communities. As members of the grid-based governance system, property managers, owners committees, and community public health service centers are responsible for assisting neighborhood committees in delivering public services. Therefore, these stakeholders are easily mobilized by neighborhood committees to participate in implementing CVCCs (ID 2).

To stimulate community volunteers to join health worker networks, neighborhood committees massively advertised the importance and urgency of the COVID-19 vaccination and volunteerism and altruism via diverse media channels, such as community bulletin boards, WeChat public accounts, and TikTok (ID 22–25). Additionally, CCP organizations at all levels required their members to actively join health worker networks in their communities to assist neighborhood committees in implementing CVCCs (ID 26–29).

#### 3.2.3 Training community health workers

Most health workers, composed of non-professionals, did not possess abundant scientific knowledge, so they failed to deliver accurate information about the COVID-19 vaccination to residents. Meanwhile, health workers who lacked proper communication skills and failed to collaborate with other stakeholders reduced the effectiveness of vaccine communication activities. Specialized training was thus expected to enhance health workers’ knowledge of vaccination, and boost their skills in collaboration and communication with residents (ID 5–8). A secretary of the neighborhood committee argued the following:

Many health workers did not have professional knowledge of vaccines, and were unclear about the safety, efficacy, procedures and precautions of COVID-19 vaccination, failing to respond to residents’ inquiries accurately. That reduced public trust in CVPs, and a professional training on these health workers is imperative (ID 7).

In response, CVP managers enhanced health workers’ communication skills via strategies such as routine professional training conducted by health experts, practical guidance, and online education programs. Specifically, neighborhood committees invited local public health experts to conduct COVID-19 vaccination training and offer practical guidance to ensure that health workers had the knowledge and skills required to implement CVCCs (ID 19, 20). Neighborhood committees also organized health workers to participate in COVID-19 vaccination online education seminars held by the district office to deepen their understanding of the laws and regulations surrounding COVID-19 vaccination to promote standardized policy implementation. This also allowed them to acquire the techniques to interact with residents. These training activities helped deter the illegal administration of CVPs and promote the efficiency of COVID-19 vaccine communication (ID 14, 15).

#### 3.2.4 Implementing media promotion

Media promotion, defined by Kabakama et al. (24) as a one-way communication approach, refers to leveraging the power of popular media tools to achieve marketing goals. Media promotion helps massively in disseminating positive information regarding a product or a behavior, to create a favorable information context and alter target populations’ attitudes and behaviors in a desirable way. Neighborhood committees, managers of community CVPs, primarily advertised COVID-19 vaccination via online and offline platforms. On the one hand, neighborhood committees hung up banners, posted on community propaganda boards, and distributed pamphlets to residents in communities to ensure widespread awareness of the importance and urgency of COVID-19 vaccination and enhance their vaccine acceptance (ID 27–30). A resident we interviewed indicated that,

Banners hung in communities claimed that: To protect your family members, please get fully vaccinated; COVID-19 vaccination benefits other people as well as oneself; vaccination helps construct herd immunity (ID 30).

On the other hand, neighborhood committees utilized multiple social media tools, such as WeChat groups, WeChat public accounts, TikTok, WeChat videos, and Sina Weibo, to massively advertise COVID-19 vaccines and ensure that residents were exposed to a huge amount of positive information related to COVID-19 vaccines, thereby increasing their willingness to get vaccinated (ID 49, 50).

#### 3.2.5 Communications between health workers and residents

COVID-19 vaccine communications between health workers and residents in the neighborhood involve persuasive, coercive, explanatory, public opinion-steered, and stakeholder collaborative patterns.

##### 3.2.5.1 Persuasive communication

Persuasive communication centers on confirming what most appeals to target audiences and then adopting tailored tactics to convince them of something. During the CVCC, the persuasive approach played an indispensable role in addressing vaccine hesitancy, and it involved logical and empathic models. The logical model highlights the use of facts, accurate evidence, and logical reasoning to create persuasive messages. In the CVCCs, health workers quoted expert opinions, statistical data, and clinic trial data to increase the credibility of arguments, curb misinformation and increase residents’ rational perception of vaccination, thereby easing vaccine concerns (ID 38–41). A doctor in the community public healthcare center noted that,

To dispel public concerns that COVID-19 vaccinations may cause leukemia and diabetes, l explained: The domestically produced vaccine is safe and has been verified by international official organizations. Meanwhile, clinical monitoring and statistical data show that in the four years before and after the COVID-19 pandemic, the number of visits and hospitalizations for diabetes and leukemia has not significantly changed, indicating that COVID-19 vaccination has not yet caused leukemia and diabetes (ID 40).

Nonetheless, the logical approach, often involving a high degree of assertiveness and aggressiveness, is not effective in all situations. Complementing the logical approach, the empathetic model also plays a critical role in persuading target people to get vaccinated. Empathetic persuasion refers to listening to target audiences’ narratives, understanding their feelings, supporting their perspectives, and reassuring their concerns in an empathetic way for behavior change. During such interactions, health workers attentively listened to residents’ narratives, shared their perspectives with residents in an empathetic way, and sincerely recommended that they get vaccinated to protect their personal health. This approach resonated with residents and enhanced public trust in CVPs, thereby decreasing vaccine hesitancy (ID 32–37). In fact, health workers employed hybrid strategies to communicate with residents about vaccine concerns instead of using a singular approach, given their respective pros and cons (ID 5).

##### 3.2.5.2 Explanatory communication

Explanatory communication, similar to question-and-answer format communication, entails that utterers respond to specific questions raised by audiences in detail to deepen their understanding of something. During the vaccine communication efforts, health workers primarily employed the explanatory model to interact with residents cautious about the COVID-19 vaccination. This approach helped increase health workers’ responsiveness to public demands, deepen residents’ understanding of the necessity, safety, efficacy, and procedures of the COIVD-19 vaccination, and eventually gain residents’ trust in CVPs. In communities, health workers mainly responded to residents’ concerns about the safety, efficacy, procedures (e.g., walk-in sites, working hours, and appointments), necessity, contraindications, possible side effects, and precautions of COVID-19 vaccination via diverse media platforms such as WeChat groups, telephone, and community temporary vaccination sites. They also answered questions from residents with limited mobility in their homes (ID 45–50). For instance, one respondent we interviewed narrated that there were over 7,000 residents in my community, disabling health workers from responding to everyone’s questions offline. As such, we established more than 100 WeChat groups to answer residents’ questions about COVID-19 vaccinations. During peak hours, each health worker answered at least 400 questions from residents every day (ID 46).

##### 3.2.5.3 Coercive communication

Coercive communication, a means of communication that exerts pressure on target audiences, members implies the adverse consequence of non-compliance to force them to act in the direction desired by the utterer. Coercive strategies are primarily applied in industries with extensive safety or operational regulations, such as the manufacturing and medical industries, to ensure that employees follow rules and stay safe, to decrease employee deviation, and to increase productivity (25). Facing top-down political pressure and influenced by the traditional governance idea of resorting to forces after courteous measures fail, some neighborhood committee members responsible for allocating community public resources probably employed coercive tactics, such as implicitly or explicitly threatening that vaccine refusers would only be able to access limited portions of community medical care facilities, year-end benefits, and educational resources, to compel them to get vaccinated after persuasive tactics failed (ID28-31). A resident we interviewed argued that,

The medical insurance, pension and year-end benefits of vaccine refusers were canceled by some neighborhood committees. Meanwhile, vaccine refusers’ children were forced to delay school enrollment (ID 31).

Coercive measures encouraged vaccine refusers to get vaccinated to a certain extent. Nonetheless, this approach violated the voluntary vaccination policy and incurred negative media reports, public criticism, mistrust in local CVPs, and public accountability for health workers who were exposed by the media to impose coercive measures. Given the pros and cons of coercive tactics, most health workers were cautious about this approach (ID 4).

##### 3.2.5.4 Public opinion-steered communication

Public opinion-guided communication is a means of communication that follows a public opinion event that emerges on social media. Media regulators control and steer the flow of public opinions in line with their governance values and expectations to avoid trust-destroying events and elicit positive sentiment expressions on social media (26). During CVCCs, health workers also emphasized managing public opinions in communities to erase citizens’ negative perceptions of COVID-19 vaccines (ID 5). In the self-media era, everyone can be a producer and disseminator of information, causing a large amount of unverified information to be disseminated on social media. The dissemination of negative vaccine information increased residents’ vaccine concerns in online communities. For instance, vaccine concerns expressed in WeChat groups involve that domestically produced vaccines are unsafe; mutations in the virus make vaccines ineffective; and vaccination induces leukemia and cancer (ID 7).

In such a case, health workers engaged in evidence-based interactions with residents, disseminated scientific evidence via diverse media tools, such as WeChat public accounts and official websites, and forwarded them expert opinions via WeChat groups to mitigate negative sentiments toward vaccination. They also advertised the hazards of rumors and the benefits of trust in science for personal health to enhance vaccine trust (ID 8–15). A health worker we interviewed argued that to allay public concerns about the effectiveness of domestically produced vaccines, we cited the results of clinical trials: Sinovac vaccine offers 64–75% protection for older adults, and Sinovac boosters have increased the protection to 98% (ID 10).

##### 3.2.5.5 Stakeholder collaboration model

Stakeholder collaboration persuasion refers to the idea that multistakeholders work together to interact with target audiences to alter their’ attitudes and behaviors in a desired way. According to Honora health workers with high charisma and social influence, extensive professional knowledge, and strong communication skills were more likely to persuade refusers to get vaccinated (27). Merely relying on a single stakeholder cannot successfully convince vaccine hesitant to get vaccinated. Connecting with health workers with different knowledge, skills, resources, and relationship networks, such as medical professionals, clinicians, neighborhood committee leaders, local celebrities, and acquaintances of target people, to jointly persuade residents to vaccinate is thus expected. In practice, the leaders of the neighborhood committee worked with doctors at the community healthcare center, acquaintances of residents who refused to get vaccinated, and so on to urge refusers to get vaccinated (ID 8–10). This stakeholder collaborative approach to vaccine communication was demonstrated to be effective (ID 9).

### 3.3 Risks in community CVCCs

According to Wang, Chinese CVCCs underpinned by the principle of “informed, consented and voluntary” that connects institutional efficiency and humanity have dispelled citizens’ misconceptions about vaccines, refuted rumors, increased the public’s scientific knowledge about vaccines, and boosted the public willingness to vaccinate (28). However, risks of CVCC have been identified, such as stigmatizing vaccine refusers, poor communication, insufficient stakeholder collaboration, and low trust in state-sponsored CVCCs.

#### 3.3.1 Stigmatizing vaccine refusers

Some neighborhood committee members constructed moral norms, promoting collectivism and community spirits, to mobilize residents to get vaccinated. Vaccine refusers were criticized by health workers as selfish, immoral, without a sense of social responsibility, violating community conventions jointly created by residents, and endangering collective security. Vaccine refusers’ medical care, pension, and year-end benefits were canceled by neighborhood committees, and property managers limited their ability to enter and exit the community freely (ID 29, 30). One of the our respondents argued that,

Some community health workers advertised that immunization is the greatest contribution to the family and the country; those who not vaccinate are selfish and immoral (ID 30).

Meanwhile, affected by cyber-nationalism and patriotism, some health workers believed that vaccination helped prevent virus spread in the country and ensure the stability and security of the country (ID 37). Those who were not vaccinated are treated as unpatriotic and as requiring punishment. Stigmatizing vaccine refusers and moral hijacking, which refers to occupying the moral ground to condemn someone and to dictate what others should do, sparked media coverage and public outrage, inducing distrust in local CVPs (29).

#### 3.3.2 Lack of transparency in communication

A lack of open communication means that health workers did not properly respond to tricky questions raised by residents, such as what are the negative effects of vaccination, why foreign-produced vaccines are not allowed, hindering the diversity of vaccine choices in China, and whether COVID-19 vaccinations were still effective as the virus mutated (ID 49, 50). A respondent elucidated that,

Vaccination’s principle is to implant the virus into the human body. While vaccination could develop immunity, it has also negative effects on human body. However, health workers convey these negative messages to citizens (ID 49).

The reasons for the lack of transparent COVID-19 vaccine communication are that health workers’ limited expertise hinders them from professionally responding to residents’ questions. Additionally, to construct the political discourse surrounding COVID-19 vaccination, grassroot-level health workers were required by governments to avoid responding to politically sensitive questions and involving themselves in topics prone to raising public controversies (ID 38–40, 51–53).

#### 3.3.3 Insufficient collaboration among health workers

Although multiple health workers banded together to a certain extent to conduct CVCCs, multistakeholder collaboration was insufficient, decreasing CVCCs’ efficiency. In practice, health workers were used to conducting CVCCs independently and only cooperated with other health workers in special situations, such as top-down political pressure for cooperation and the failure of vaccine communication led by a single stakeholder (ID 32–37). A member of the neighborhood committee argued that,

We usually work independently, and will only collaborate with others to conduct vaccine communication required by the sub-district office, or when we failed to persuade residents to get vaccinated (ID 37).

Insufficient stakeholder collaboration is attributed to health workers’ disagreements regarding strategies for implementing CVCCs and a lack of collaboration. Health workers disagreed with the schedule and strategies for implementing CVCCs based on their availability and values, perceptions, and experiences of the COVID-19 vaccination. Failure to tackle and address these disagreements reduced health workers’ willingness to cooperate. Consequently, instead of diverse stakeholder collaboration, community health workers preferred to clearly define their respective responsibilities to facilitate the independent performance of CVCCs rather than teamwork (ID 42–49).

#### 3.3.4 Distrust in state-sponsored CVCCs

The political tendency of CVCCs and low trust in neighborhood committees caused some residents to express their distrust and even resistance to CVCCs. To complete the political tasks assigned by the higher state and obtain a good performance appraisal, neighborhood committees adopted various measures to endlessly pressure residents to ensure compliance, arousing public disgust (ID 24–28). A resident argued that,

Neighborhood committees competitively conducted vaccination campaigns to achieve a high vaccination rate and get rewards from senior governments rather than to serve the people and protect personal health (ID 25).

Low trust in neighborhood committees also bred distrust in community CVPs. Neighborhood committees, although legally deployed to represent and serve public interests in communities, function as an extension of the government apparatus in reality to implement administrative tasks assigned by states and are less responsive to public demands. The bureaucratisation of public service provision, neighborhood committees’ weak sense of serving the people, and unfair public resource distribution have greatly reduced public trust in the neighborhood committee. Meanwhile, members of neighborhood committees lack vaccination-related expertise, reducing the credibility of CVCCs initiated by neighborhood committees. During the CVCCs, neighborhood committees primarily marketed the safety, necessity, and urgency of COVID-19 vaccination to mobilize residents to get vaccinated. However, they failed to answer residents’ questions professionally, arousing public skepticism.

To ensure the continuity of CVCCS in modern China, health workers are expected to maintain more open communication with residents and be inclusive of vaccine refusers. Furthermore, CVP managers should create robust partnerships among health workers by ensuring their agreement on strategies for implementing CVCCs and optimize COVID-19 immunization service delivery to depoliticize community CVPs.

## 4 Discussion

**Figure 1:**
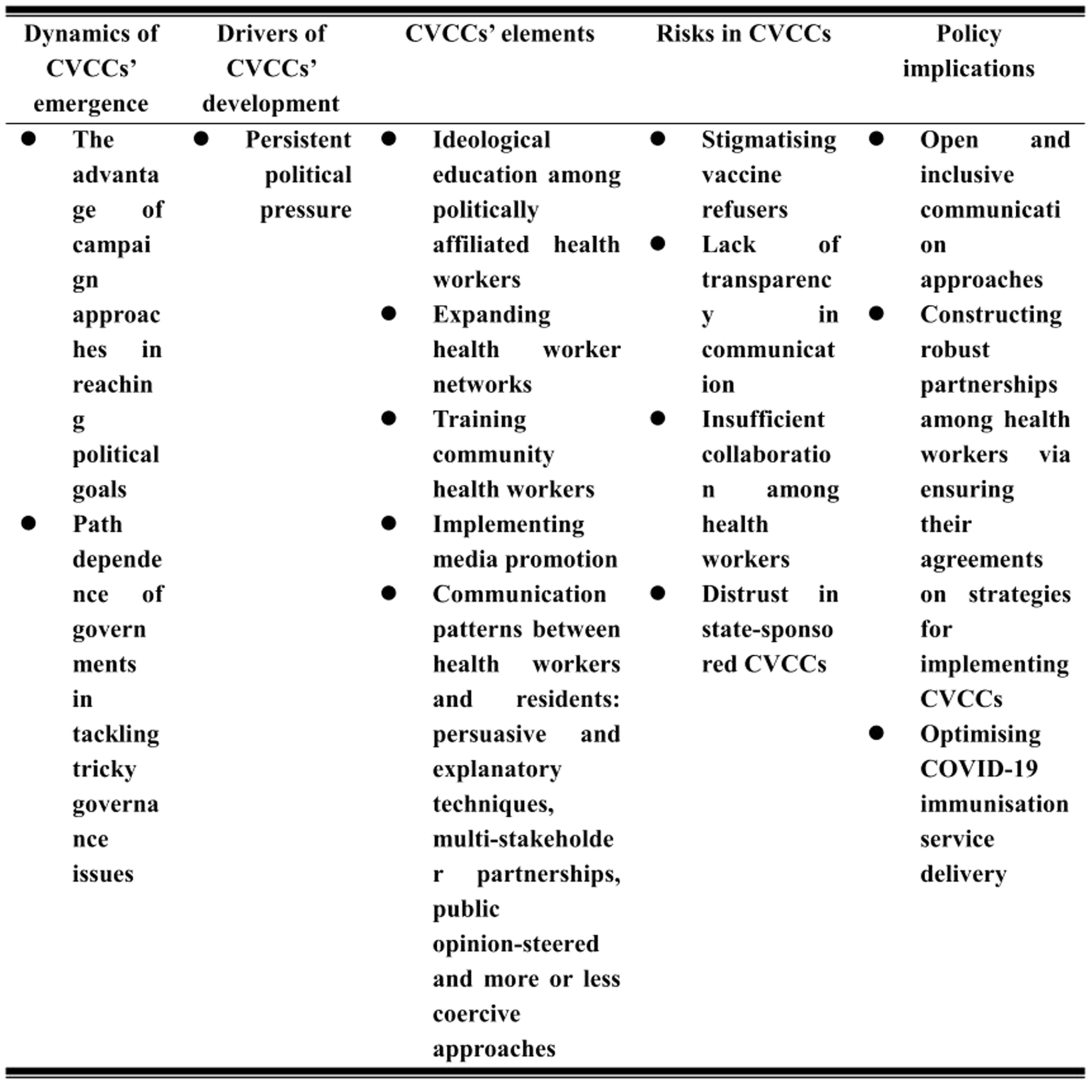
Organization of CVCCs in Chinese communities.

This analysis shows that CVCCs were driven by top-down political pressure. The components of CVCCs involved ideological education among politically affiliated health workers, expanding health worker networks and training health workers, communicating with residents using persuasive and explanatory techniques, encouraging stakeholder collaboration, and using public opinion-steered and coercive approaches. While CVCCs significantly enhanced COVID-19 vaccine acceptance, a lack of open communication, the stigmatization of vaccine refusers, and low trust in CVCCs eroded CVCCs’ validity. To promote the continuity of CVPs in modern China, community health workers must communicate with residents in a more open and inclusive way. Furthermore, CVP managers should create robust partnerships among health workers by ensuring their agreements on strategies for implementing CVCCs and optimizing COVID-19 immunization service delivery to depoliticize CVPs (Figure 1).

COVID-19 vaccine hesitancy has raised scholarly attention to COVID-19 vaccine communication efforts. Extant research illustrates the principles and strategies of COVID-19 vaccine communication (30). COVID-19 vaccine communication principles involve transparency, intelligibility, and consistency of information delivery and the inclusiveness of interaction to avoid negative assumptions or stereotyping associated with ethnicity (31, 32). COVID-19 vaccine communication tactics include rational persuasion, emotional appeals, message framing, A social marketing mix, contextualized models, mass media campaigns, construction of trusted networks, and stakeholder collaboration. Rational persuasion highlights that health workers provide credible information such as statistical data, clinical trial data, and expert opinions to address citizens’ specific concerns, neutralize misinformation, and strengthen vaccine-supporting voices (1, 33, 34). Emotional appeals entail that health workers empathetically respond to vaccine concerns and strengthen individuals’ moral norms to raise vaccination intention (35–37). Message framing includes gain-framed and loss-framed messages. The former highlights the benefits of adopting a recommended behavior, while the latter underlines the losses stemming from not adopting a recommended behavior. Given this, health practitioners must communicate the societal and individual benefits of COVID-19 vaccinations and the risks of vaccine refusal to citizens (35, 38). The social marketing mix, demonstrated by Hong as an effective COVID-19 vaccination approach in South Korea, involves product communication, highlighting the community and individual benefits of vaccination; price communication, referring to the drivers of COVID-19 vaccination such as self-efficacy, personal health, and rewards; place communication, denoting informing residents timely of the schedule and sites of COVID-19 vaccination service delivery; and promotion communication, entailing delivering clear, accurate, and coherent information via trusted media outlets (39). Constructing a trusted network helps incorporate influencers of vaccination decisions, such as medical professionals, celebrities, opinion leaders, and acquaintances, into CVCCs to deliver pro-vaccine messages and enhance public trust in COVID-19 vaccines (32, 40). Reinforcing partnerships among health workers supports connecting various stakeholders’ knowledge, experience, and resources and increases the accuracy and receptivity of information delivered. Therefore, Gao et al. (41) propose creating collaborations involving local governments, doctors, and universities to communicate with vaccine-hesitant students. Chou et al. (1) also suggested a contextualized communication model tailored to a community’s culture, values, concerns, and information needs. Similarly to previous studies, our study also highlighted the efficacy of reliable collaboration networks, rational persuasion, emotional appeals, and coordinated COVID-19 vaccination communication approaches. Building upon previous studies, our study revealed the political context of CVCCs in China, ideological education among politically affiliated health workers, coercive and public opinion-steered communication tactics, and the risks of CVCCs in an authoritarian regime. Interestingly, our analysis indicated that CVCCs exhibited a stress-response pattern in China. The CVCC was sustained by political forces and was significantly affected or even disrupted by top-down political pressure. In this context, community health workers were less responsive to public concerns about vaccines and increased vaccine hesitancy. To achieve a high vaccination rate, top-level governments had to continuously exert political pressure on grassroot-level governments to sustain CVCC practices in China.

The Chinese government deployed the mobilization campaign to roll out CVPs across the country to reach herd immunity. The central state conceptualized COVID-19 vaccination as a “major political task,” enacting political pressure and ideological education and creating accountability and incentive systems to promote the political loyalty of local officials and ensure that they actively performed their duties (42). Local states, based on the principle of handling special matters with special arrangements, prioritized vaccination tasks, tweaked the bureaucratic system, and accumulated social capital, aiming to achieve a high vaccination rate swiftly. At the neighborhood level, health workers advertised collectivist values and socialist morality, oriented public opinions, and constructed favorable information contexts to raise public acceptance of the COVID-19 vaccination. They coercive adopted deterrent strategies to compel public obedience (43). This reflects the institutional characteristics of CVCCs in authoritarian China. Although vaccine mobilization practices significantly increased Chinese COVID-19 vaccination rates, the inappropriate organization of CVCCs eroded the legitimacy of vaccinations. Politicization of CVCCs bred distrust in CVPs, local coercive styles of vaccine communication induced media exposure and public criticism, and vaccine communication activities underpinned by collectivism and patriotism stigmatized vaccine refusers. Therefore, counteracting the political tendencies of and reconciling government–market–society forces in immunization campaigns in modern China warrants further research.

### 4.1 Limitation of this research

This qualitative study deepened our understanding of CVCCs in Chinese grassroots society. Nonetheless, this research must be considered against the background of its limitations. First, we selected participants using an informal and snowballing approach instead of a scientific sampling method to align with China’s highly relationship-oriented society (44). However, data gleaning by snowballing in this study conformed to the principle of data saturation. Second, although two professionals are involved in reviewing each phase of data analysis, a more rigorous thematic analysis (e.g., researcher triangulation, description of audit trails, peer debriefing, and member checking) is expected to be conducted to establish trustworthiness in qualitative research. Finally, this study centers on CVCCs in urban communities, but not yet on CVCCs in rural China. According to Zhao, China’s urban–rural divide, with a focus on constraining rural-to-urban mobilization by a household registration system instituted by the government in 1958 and differentiated resource input between rural and urban areas, induced urban–rural differences in the contexts, strategies, and risks of COVID-19 vaccine communication activities (45). Future research is thus expected to explore the CVCC in rural China.

## 5 Conclusion

The continuity of CVCCs was driven by top-down political pressure. The components of CVCCs involve conducting ideological education among politically affiliated health workers, expanding health worker networks, training health workers, implementing media promotion, communicating with residents using persuasive and explanatory techniques, encouraging stakeholder collaboration, and using public opinion-steered and coercive approaches. While CVCCs significantly enhanced COVID-19 vaccine acceptance, inadequate openness in communication, stigmatizing vaccine refusers, insufficient stakeholder collaboration, and low trust in CVPs eroded CVCCs’ validity. To favor the continuity of CVCCs in China, CVCC performers are expected to conduct more open and inclusive communication with residents. Furthermore, CVP planers should also create robust partnerships among health workers by ensuring stakeholders’ agreements on strategies for implementing CVCCs and optimize COVID-19 immunization service provision to depoliticize CVPs.

## Data availability statement

The raw data supporting the conclusions of this article will be made available by the authors, without undue reservation.

## Ethics statement

Ethical approval was not required for this study. The studies presented in this article were conducted in accordance with local legislation and institutional requirements.

## Author contributions

RY: Investigation, Resources, Data curation, Formal analysis, Methodology, Writing – original draft, Funding acquisition. YH: Conceptualization, Methodology, Formal analysis, Writing – review & editing, Supervision, Validation, Visualization.
